# Superior Mesenteric Artery Stenosis Presenting as Chest Pain: Danger in Disguise

**DOI:** 10.7759/cureus.50922

**Published:** 2023-12-21

**Authors:** Satish Mahajan, Nikhil Pantbalekundri, Kashish Khurana, Ajinkya Kadu

**Affiliations:** 1 Department of Medicine, Jawaharlal Nehru Medical College, Datta Meghe Institute of Higher Education and Research, Wardha, IND

**Keywords:** postprandial angina, coronary artery disease, acute intestinal ischemia, mesenteric artery stenosis, chronic mesenteric ischemia

## Abstract

Chronic mesenteric ischemia (CMI), often known as abdominal angina, is a syndrome caused by a severe reduction in arterial flow to the digestive loops. It is an uncommon and underdiagnosed entity with potential severe adversities, such as acute mesenteric ischemia (AMI). Patients with coronary artery disease (CAD) are shown to also have mesenteric artery stenosis (MAS). By identifying risk variables, it may be possible to screen for mesenteric artery involvement in patients with CAD who exhibit an elevated risk. Here, we present a unique case of a person with severe retrosternal chest pain with postprandial angina, which turned out to be superior mesenteric artery (SMA) ostial stenosis.

## Introduction

Intestinal ischemia is brought on by mesenteric artery stenosis (MAS), which restricts blood supply to the small intestine. Atherosclerosis is typically the cause of chronic mesenteric ischemia (CMI); trauma and severe fibromuscular illness are unusual causes [1]. The inferior mesenteric artery (IMA), superior mesenteric artery (SMA), and celiac trunk frequently have ostial disease, where proximal few centimeters of these arteries frequently have occlusions. At least two of the three primary splanchnic arteries must have substantial stenosis in order to cause "chronic mesenteric ischemia" [2]. Symptomatic cases generally involve the SMA. Patients have enough intestinal blood flow while they are at rest to keep their gastrointestinal tract viable and stop the onset of symptoms. However, the increased demand on the mesenteric circulation following a meal may outweigh the collateral circulation's capacity to compensate, leading to postprandial intestinal angina. Most chronic mesenteric ischemia patients first exhibit symptoms in their fifth or sixth decade of life. Females are more prone than males according to a study [3]. They frequently have other forms of atherosclerotic disease, such as coronary artery disease (CAD), cerebrovascular disease, or peripheral vascular disease. Postprandial abdominal discomfort is the most common complaint among most patients. The gastrointestinal symptoms of CMI are non-specific, which makes it challenging for a physician to distinguish between CMI and other more prevalent diseases, such as CAD, as presented in the following case.

## Case presentation

A 52-year-old male presented to the emergency department with a one-day duration retrosternal chest pain radiating to the back. The pain was insidious in onset and progressive in nature. It was exacerbated by food consumption; however, it was not associated with breathlessness, palpitations, diaphoresis, nausea, vomiting, or loose stools. His symptoms were refractory to intravenous pantoprazole 40 mg and antacids; hence, he was referred to our center by his family physician. The patient has had similar episodes in the past three months when he did not seek any medical help as it was relieved on resting. He has a past medical history of hypertension since 10 years, which was managed with telmisartan 40 mg.

On examination, the patient was conscious, uncomfortable, and restless in bed. The blood pressure was 140/90 mmhg, and the pulse rate was 110/minute. On auscultation, his chest was clear. The abdominal examination was insignificant. Routine blood tests were reported within normal range. Chest X-ray was suggestive of no significant abnormality. Electrocardiogram (ECG) was suggestive of tall QRS complexes in V5 and V6 with no significant ST-segment or T changes. Due to acute coronary syndrome suspicion, cardiac enzymes were sent. However, they were within normal limits.

**Figure 1:**
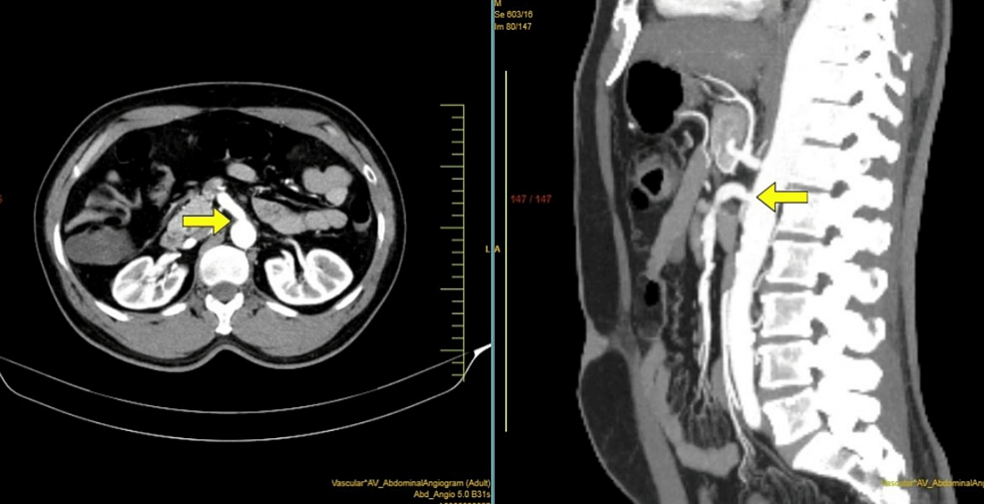
CT aortogram displaying superior mesenteric artery ostial stenosis (yellow arrow)

**Figure 2:**
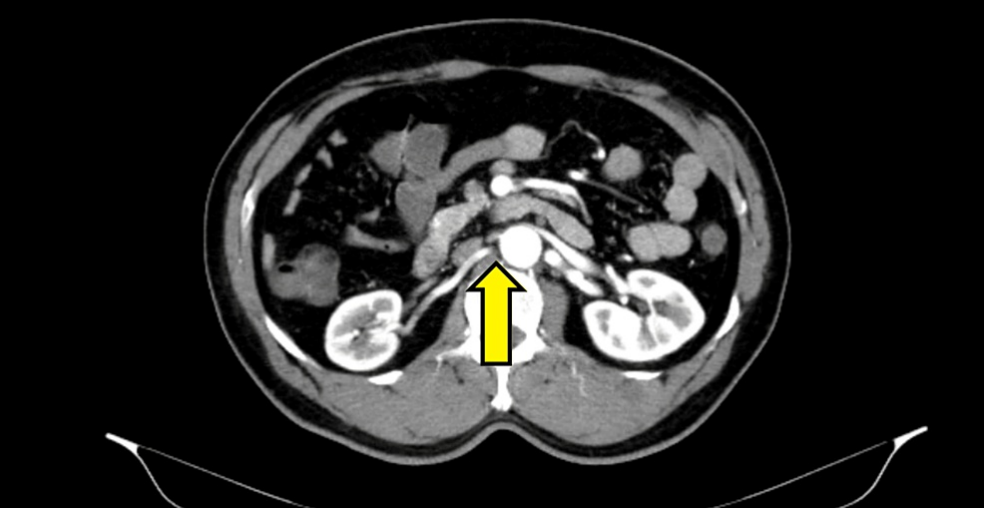
CT aortogram displaying right renal artery stenosis (yellow arrow)

A 2D echocardiography (2D-echo) was done, which was suggestive of mild concentric left ventricular hypertrophy but no regional wall motion abnormality. With poor evidence to support CAD, a computed tomography (CT) of the aorta and its branches (CT-aortogram) was done with suspicion of aortic dissection. It revealed significant (more than 70%) superior mesenteric artery ostial stenosis (Figure 1) with right renal artery stenosis (Figure 2).

**Figure 3:**
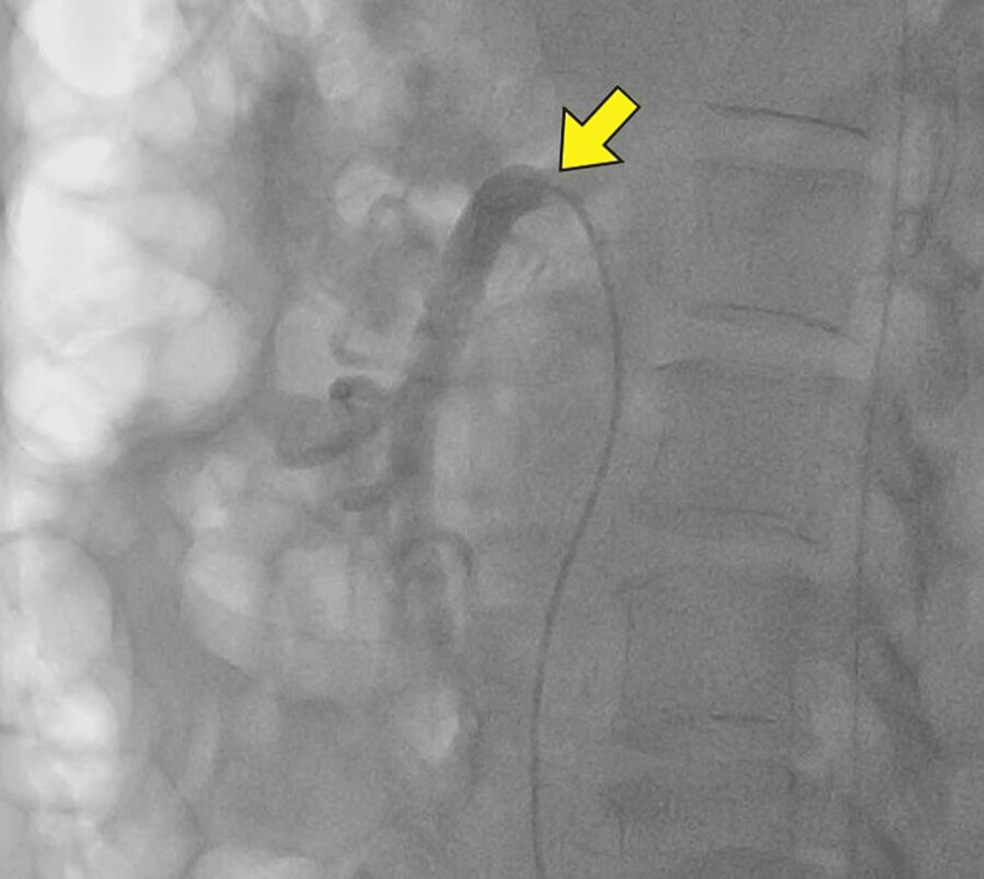
Fluoroscopy demonstrating superior mesenteric artery ostial stenosis (yellow arrow)

**Figure 4:**
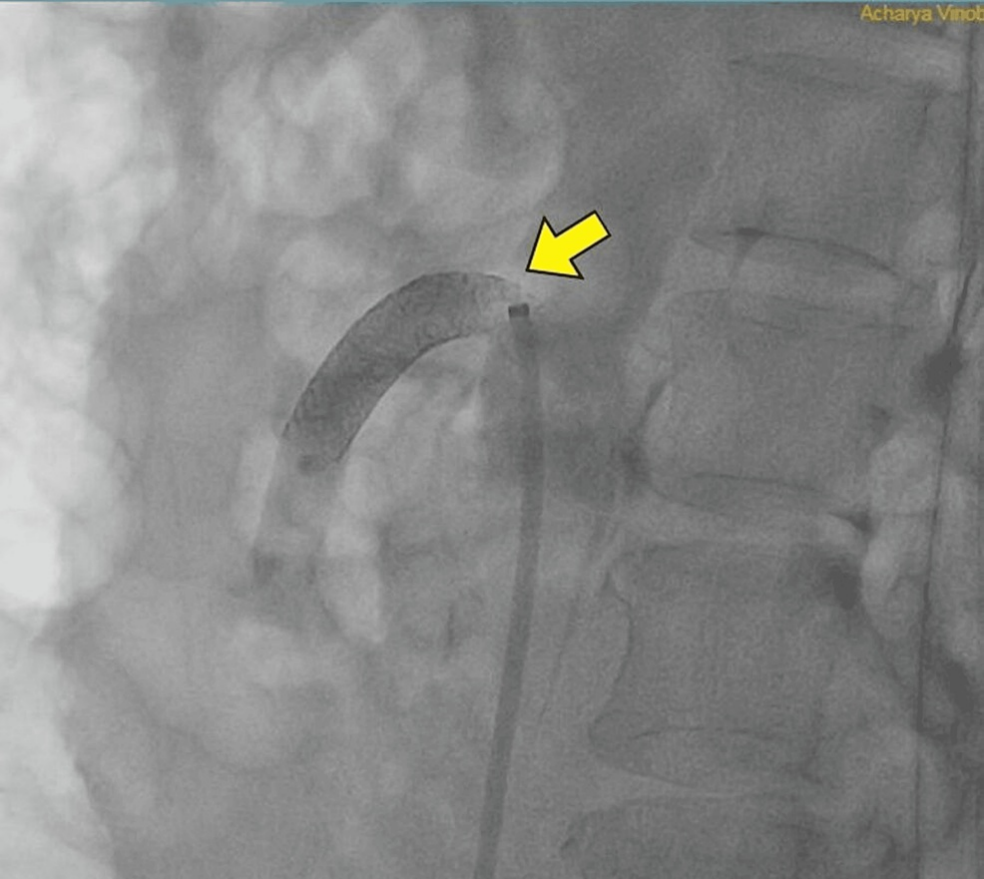
Fluoroscopy demonstrating flow after stenting of superior mesenteric artery (yellow arrow)

**Figure 5:**
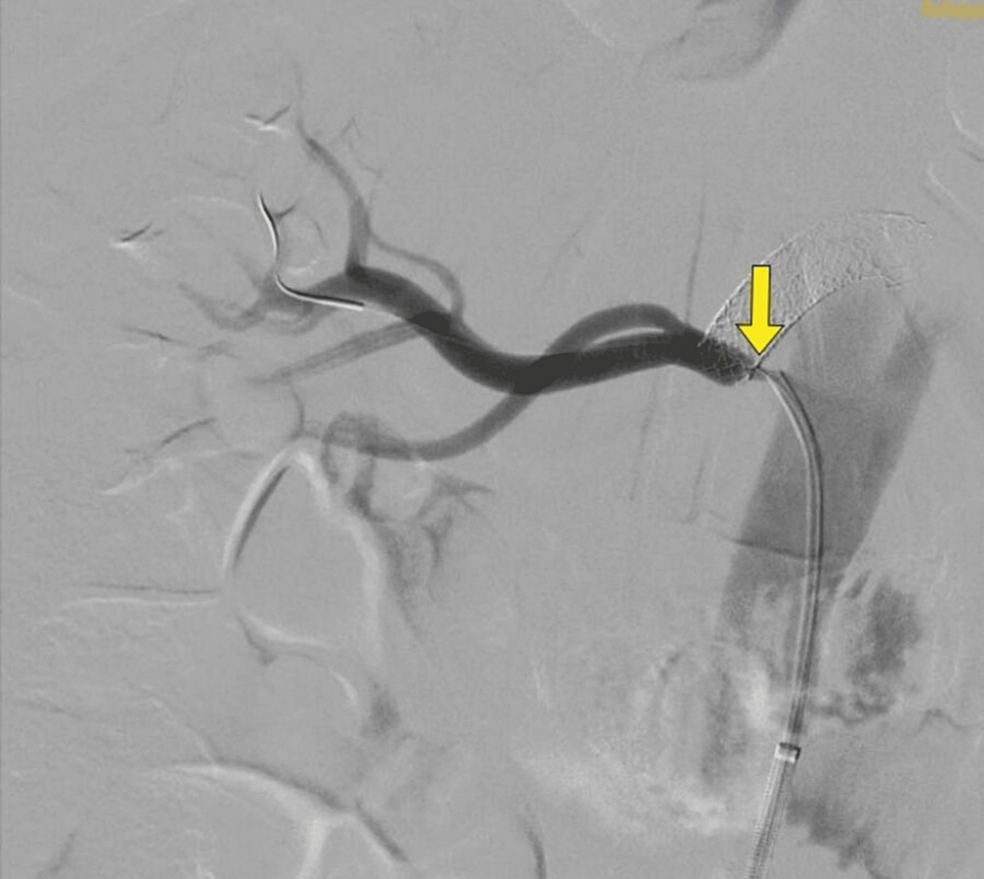
Fluoroscopy demonstrating right renal artery ostial stenosis (yellow arrow)

**Figure 6:**
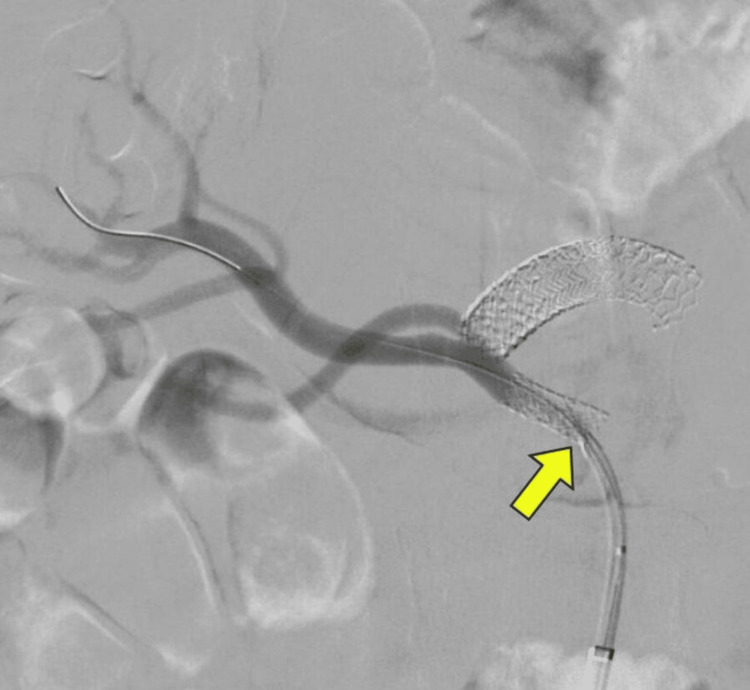
Fluoroscopy demonstrating flow after stenting of right renal artery (yellow arrow)

Interventional radiologist opinion was obtained, and fluroscopic imaging was done. Superior mesenteric ostial stenting (Figures 3, 4) and right renal artery stenting (Figures 5, 6) were done. Post-stenting patient was symptomatically relieved and started on anti-platelets and statins. On follow-up, the patient did not have further episodes.

## Discussion

Due to severe mesenteric artery stenosis, mesenteric ischemia, also known as intestine angina, manifests itself in a variety of ways, generally affecting elderly people. Despite advances in understanding of mesenteric ischemia, diagnostic techniques, and available therapies, recognizing the condition is still complex. This results in treatment implementation being dragged, which raises mortality and morbidity rates. Occlusive mesenteric artery ischemia (OMAI) and non-occlusive mesenteric ischemia (NOMI) are the two basic clinical entities that make up acute mesenteric ischemia (AMI). Acute mesenteric arterial embolism (AMRE) and acute mesenteric arterial thrombosis (AMRT) are further subtypes of OMAI. Mesenteric venous thrombosis (MVT) is the venous illness caused by AMI. AMI affects 0.1% of all hospital admissions overall, and 0.001% of patients who have exploratory laparotomies have venous thrombosis [4]. Arrhythmia, mitral stenosis, endocarditis, recent myocardial infarction, and intra-abdominal cancers are risk factors for AMI [5].

Any degree of vascular narrowing in the mesenteric region that is associated with at least one of the following conditions is known as clinically relevant mesenteric artery stenosis (CR-MAS): 1) the existence of classic mesenteric angina with any level of MAS and 2) significant vascular stenosis affecting two or more vessels [6]. The following are the categories of MAS: Stenosis is considered normal when it is less than 30%, mild stenosis is defined as any degree of narrowing that does not meet the criteria for severe stenosis, and severe stenosis is defined as any of the following: a narrowing of at least 70%, any degree of narrowing with post-stenotic dilatation, or the presence of collaterals.

Vascular lesions are frequently found when a different clinical disease is being evaluated in such patients. Thus, a physician may detect mesenteric arterial stenosis or occlusion without any symptoms. There are minimal natural history data available for the clinical course of asymptomatic diseases involving the major mesenteric arteries in comparison to carotid, coronary, renal, and peripheral vascular diseases [7]. Estimates of the prevalence of mesenteric illness based on autopsies range from 30% to 80% [8]. However, population-based data are limited. Angiographic case series have identified relationships between symptomatic peripheral atherosclerosis and renal atherosclerosis with undetected mesenteric artery lesions [9,10]. A recent population-based research found that 17.5% of an elderly, free-living cohort had asymptomatic celiac axis (CA) or superior mesenteric artery (SMA) stenosis at duplex ultrasound (US) [11,12]. Only 2.5% of the participants had SMA disease, although it was linked with weight loss and renal artery stenosis [13].

In a study after treating CAD, individuals with CR-MAS were evaluated. Using a thorough history and clinical examination, the mesenteric arteries were re-evaluated (using CT or Doppler US) during follow-up. Patients with chronic CAD were found to have a high prevalence of MAS. Patients who refused intervention had medical care (lifestyle medication, comorbid disease control, antiplatelet, and statins) [14]. Lifestyle modification and medication for atherosclerotic ischemic heart disease probably prevents AMI in CAD patients. However, patients with CR-MAS were suggested to undergo endovascular intervention or surgery if their abdominal anginal symptoms persisted despite medication therapy for six months [15].

## Conclusions

Although severe stenosis of at least two vessels between the celiac and mesenteric arteries is the standard definition of intestinal angina, the focus of this case is on the evaluation and necessity for a complete evaluation of the patient who is presenting with angina for mesenteric ischemia. To rule out an acute mesenteric infarction, cases of AMI should be examined using CT-angiography or, if possible, arteriography. Early diagnosis is hampered by a lack of credible non-invasive testing that can reliably diagnose mesenteric ischemia. The potential identification of MAS risk factors in CAD patients may aid in risk assessment and direct suitable therapy.
